# Cardiovascular and renal protective effects of non-vitamin K antagonist oral anticoagulants and warfarin in patients with atrial fibrillation

**DOI:** 10.1371/journal.pone.0275103

**Published:** 2022-10-13

**Authors:** Seong Huan Choi, Mina Kim, Hoseob Kim, Dae-Hyeok Kim, Yong-Soo Baek

**Affiliations:** 1 Inha University College of Medicine and Inha University Hospital, Incheon, Republic of Korea; 2 Data Science Team, Hanmi Pharm. Co., Ltd., Seoul, Republic of Korea; Cleveland Clinic, UNITED STATES

## Abstract

**Aim:**

Data on the use of non-vitamin K antagonist oral anticoagulants (NOACs) in relation to the risk of cardiovascular (CV) disease and renal protection among patients with atrial fibrillation (AF), are relatively sparse. We aimed to compare the effectiveness and safety of NOACs with those of warfarin for vascular protection in a large-scale, nationwide Asian population with AF.

**Methods and results:**

Patients with AF who were prescribed oral anticoagulants according to the Korean Health Insurance Review and Assessment database between 2014 and 2017 were analyzed. The warfarin and NOAC groups were balanced using propensity score weighting. Clinical outcomes included ischemic stroke, myocardial infarction, angina pectoris, peripheral artery disease, chronic kidney disease (CKD), end-stage renal disease (ESRD), CV death, and all-cause death. NOAC use was associated with a lower risk of angina pectoris (HR, 0.79 [95% CI, 0.69–0.89] p<0.001), CKD stage 4 (HR, 0.5 [95% CI, 0.28–0.89], p = 0.02), and ESRD (HR, 0.15[95% CI, 0.08–0.32], p<0.001) than warfarin use. NOACs and warfarin did not significantly differ with respect to stroke reduction (HR, 1.05 [95% CI, 0.88–1.25], p = 0.19). NOAC use was associated with a lower risk of intracranial hemorrhage (HR, 0.6 [95% CI, 0.44–0.83], p = 0.0019), CV death (HR, 0.55 [95% CI, 0.43–0.70], p<0.001), and all-cause death (HR, 0.6 [95% CI, 0.52–0.69], p<0.001) than warfarin use.

**Conclusion:**

NOACs were associated with a significantly lower risk of adverse CV and renovascular outcomes than warfarin in patients with AF.

## Introduction

Atrial fibrillation (AF) has become one of the most common cardiovascular (CV) diseases. It has shown a rapid rise in prevalence for nearly half a century [1]. A broader understanding of AF has led to improvements in the prevention of ischemic stroke, myocardial infarction (MI), and other clinical consequences [2, 3]. The use of the CHA_2_DS_2-_VASc scoring system, devised to assess the probability of ischemic stroke and oral anticoagulants, has drastically decreased the incidence of these life-threatening events among patients with AF. Over the years, many studies have been conducted to identify an effective and safe anticoagulant [4, 5]. In the early 1980s, aspirin was considered the anticoagulant of choice but was quickly replaced by warfarin owing to its greater efficacy. Further research revealed that therapeutic dose adjustment for warfarin was a hindrance in patient compliance. Moreover, warfarin is associated with an increased risk of intracranial bleeding, along with major bleeding, and these became an issue. The introduction of non-vitamin K antagonist oral anticoagulants (NOACs) has provided a platform for discussion regarding these matters, and numerous studies have directly compared the safety and efficacy of NOACs and warfarin [6]. Despite the abundance of studies on NOACs, data regarding the vascular protective effects of NOACs, including their effects on the incidence of coronary artery occlusive disease (CAOD), peripheral artery occlusive disease, and renal insufficiency in a large-scale population, are relatively sparse. Therefore, we aimed to evaluate the vascular protective effects of NOACs compared to warfarin in a large-scale, nationwide population.

## Materials and methods

### Study design and patient selection

**Fig 1:**
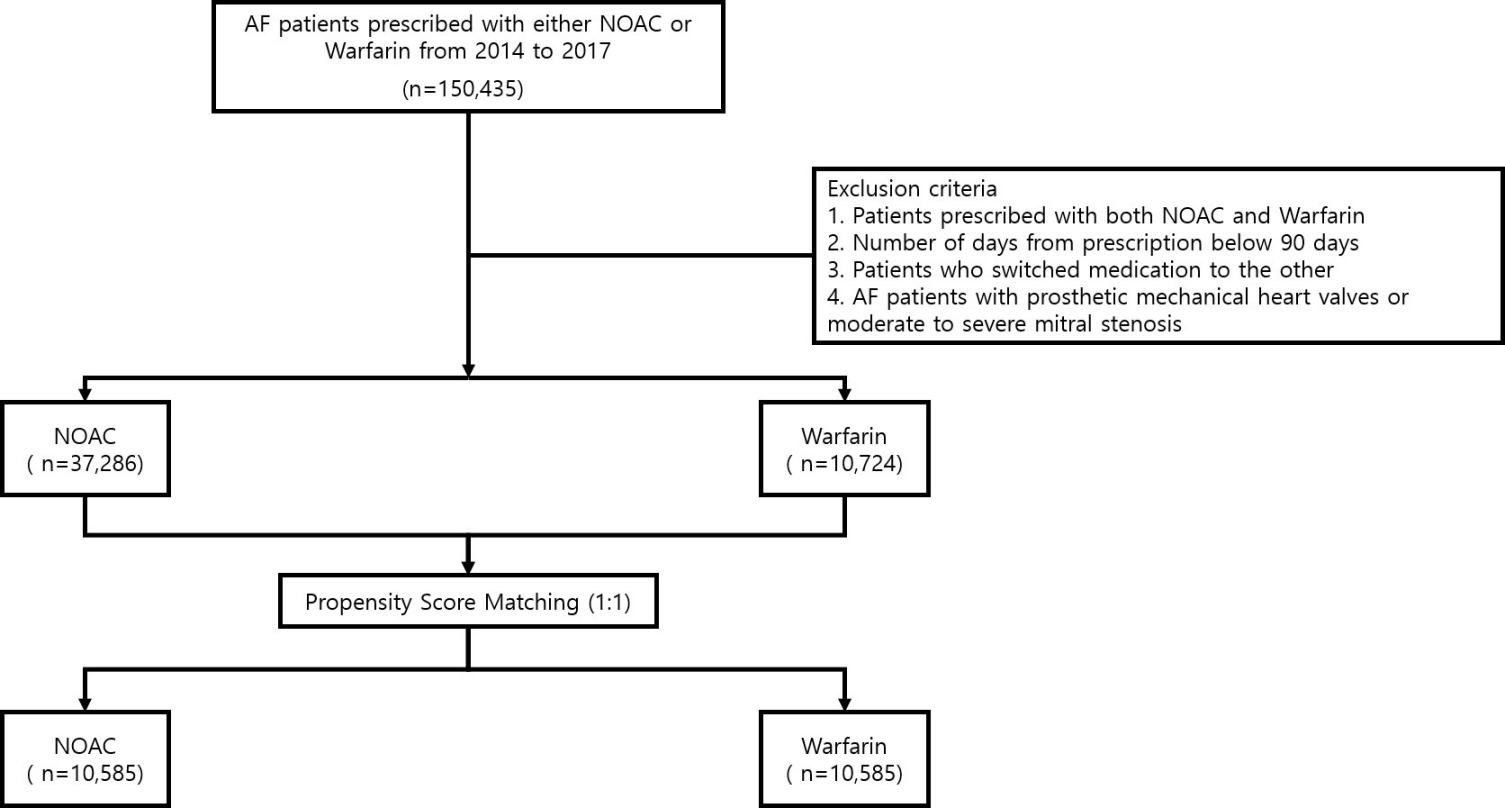
Flowchart of the study design and population.

Data were acquired from the Korean National Health Insurance Service (NHIS) database. Patients with AF were identified using codes from the International Classification of Disease, tenth revision. Patients with AF who were prescribed warfarin or NOACs between 2014 and 2017 were analyzed. Exclusion criteria included patients who were prescribed both NOACs and warfarin, patients whose oral anticoagulant usage (warfarin or NOACs) was below 90 days, patients who had switched from one medication to the other, and patients with AF with prosthetic mechanical heart valves or moderate to severe mitral stenosis. The study population was divided into the NOAC and warfarin groups (Fig 1). Medication compliance was defined as the ‘number of days of actual prescribed medication/total outpatient clinic follow-up days x100’. Values above 80% were considered good medical compliance, and values below 80% were considered poor compliance [7, 8]. The requirement for informed consent was waived by our institutional review board (2019-03-031), because each patient identified through the NHIS database was automatically de-identified to ensure personal privacy.

### Study outcomes and follow-up data acquisition

The primary outcomes of our study was ischemic stroke and all-cause mortality. Secondary outcomes were MI, angina pectoris, renal function impairment (development of chronic kidney disease [CKD] or escalation to end-stage renal disease [ESRD]), intracranial hemorrhage (ICH), gastrointestinal (GI) bleeding, CV-related death, and major adverse cardiovascular events (MACEs). MACEs were defined as the cumulative events of MI, stroke, and CV-related death.

### Statistical analysis

We conducted 1:1 propensity score matching to adjust for each group’s baseline characteristics. The absolute standardized mean difference was calculated to validate the propensity score matching. Age, sex, history of heart failure, hypertension (HTN), diabetes mellitus (DM), vascular events, ischemic stroke, transient ischemic attack, and thromboembolism were corrected for confounding factors. Continuous data were presented as mean ± standard deviation. Categorical data as percentages or absolute numbers. Continuous data were analyzed using analysis of variance, and categorical data were analyzed using the chi-square test to assess the differences between the two groups. Cox proportional hazard regression analysis utilizing the backward elimination technique was performed to analyze the effects of NOACs and warfarin on the study outcomes. Hazard ratios (HRs) were calculated as an estimate of the risk associated with a particular variable, with 95% confidence intervals (CIs). The proportional hazard assumptions of HR in the Cox proportional hazard models were graphically inspected in the “log minus log” plot and tested using Schoenfeld residuals. The omitted columns represent the multivariate parameters that were not statistically significant. The Kaplan–Meier (KM) method was used to estimate event-free survival. All analyses were performed using SPSS (version 19.0; SPSS, Chicago, IL, USA) and SAS (version 9.3; SAS Institute, Cary, NC, USA). Statistical significance was set at p<0.05.

## Results

### Baseline characteristics of the study population

Among the 48010 patients with AF, 10724 were treated with warfarin and 37286 with NOACs. A total of 10585 patients were matched and allocated to the NOAC and warfarin groups. Specific NOAC prescription rates were as follows: dabigatran (n = 1851), rivaroxaban (n = 3634), apixaban (n = 2696) and edoxaban (n = 2404)

Clinical parameters, such as age, body mass index (BMI), systolic blood pressure (SBP), diastolic blood pressure (DBP), DM, HTN, CKD, and the CHA_2_DS_2-_VASc scores were not evenly matched. Therefore, relevant factors were utilized in the Cox regression models for adjustments. The absolute standardized difference was calculated to minimize bias between the two groups. The mean patient age in the NOAC and warfarin groups was 66.23±12.43 and 65.79±13.15 years, respectively. The mean CHA_2_DS_2-_VASc score in the NOAC and warfarin groups was 3.36±1.93 and 3.30±1.99, respectively. The baseline characteristics of the study population before and after propensity score matching are shown in Table 1.

**Table 1:** Baseline characteristics of the study population.

Baseline characteristics	Total (48,010)	Before matching			After matching			
		Warfarin (10,724)	NOAC (37,286)	p value	Warfarin (10,585)	NOAC (10,585)	p value	SMD
Sex, Female, n(%)	21382(44.5%)	4032(37.6%)	17350(47%)	<0.001	4012(37.9%)	3881(36.7%)	0.06	-0.0256
Age (Mean, SD)	70.83±11.45	65.31±13.7	72.42±10.14	<0.001	65.79±13.15	66.23±12.43	0.01	-0.03472
Medical compliance					7554(71.4%)	8903(84.12%)	<0.001	-0.30999
Smoking,n(%)				<0.001			0.06	
*no*	15622(61.3%)	2886(53.9%)	12736(63.2%		2871(54.1%)	3259(54.8%)		
*ex-smoker*	6330(24.8%)	1385(25.9%)	4945(24.5%)		1379(26.0%)	1622(27.3%)		
*current smoker*	3550(12.9%)	1081(20.2%)	2469(12.3%)		1061(20.0%)	1069(18.0%)		
BMI (Mean, SD)	24.85±3.43	24.564±3.40	24.94±3.43	<0.001	24.54±3.39	25.18±3.46	<0.001	
SBP (Mean, SD)	128.67±15.86	126.9±15.87	129.14±15.82	<0.001	126.91±15.88	128.26±15.71	<0.001	
DBP (Mean, SD)	77.92±10.58	77.32±10.79	78.08±10.51	<0.001	77.31±10.78	78.61±10.75	<0.001	
Ischemic stroke,n(%)	8687(18.1%)	1962(18.3%)	6724(18%)	0.54	1952(18.4%)	2010(19.0%)	0.32	-0.01405
Diabetes mellitus,n(%)	19950(41.6%)	4167(38.9%)	15783(42.3%)	<0.001	4136(39.1%)	4347(41.1%)	0.003	0.04069
Hypertension,n(%)	37649(78.4%)	7763(72.4%)	29931(80.3%)	<0.001	7712(72.9%)	7880(74.4%)	0.009	0.03603
Ischemic cardiomyopathy,n(%)	409(0.9%)	128(1.2%)	281(0.8%)	<0.001	127(1.2%)	79(0.7%)	<0.001	0.04621
Chronic Kidney disease,n(%)	18590(38.7%)	4026(37.5%)	14564(39.1%)		4363(41.2%)	4367(41.3%)		
*Stage 1*	36(0.1%)	12(0.1%)	24(0.1%)	0.16	4(0.0%)	3(0.0%)	1	0.0052
*Stage 2*	13302(27.7%)	2373(22.1%)	10929(29.3%)	<0.001	2847(26.9%)	3526(33.3%)	<0.001	-0.14018
*Stage 3*	4185(8.7%)	842(7.9%)	3343(9.0%)	<0.001	805(7.6%)	767(7.2%)	0.33	0.01369
*Stage 4*	365(0.8%)	225(2.1%)	140(0.4%)	<0.001	137(1.3%)	26(0.2%)	<0.001	0.12018
*ESRD*	702(1.5%)	574(5.4%)	128(27.0%)	<0.001	5.4%)	45(0.4%)	<0.001	0.29858
Heart Failure,n(%)	6876(32%)				3379(32%)	3497(33%)	0.08	0.02381
Vascular disease,n(%)	12484(26.0%)	2426(22.6%)	10058(27.0%)	<0.001	2420(22.9%)	2410(22.8%)	0.88	-0.00225
CHA_2_DS_2-_VASc score								
Mean (SD)	3.8±1.85	3.27±2	3.96±1.78	<0.001	3.3±1.99	3.36±1.93	0.028	0.0302
Median	4	3	4		3	3		
*0–1*	5008	2305	2703		2211	1892		
*2–3*	16369	3601	12768		3562	3846		
*over 4*	26633	4818	21815		4812	4847		

Data are presented as means ± SDs or numbers (%).

NOAC, non-vitamin K antagonist oral anticoagulant; SD, standard deviation; BMI, body mass index; SBP, systolic blood pressure; DBP, diastolic blood pressure; ESRD, end-stage renal disease; SMD: Standardized mean difference.

### Primary outcomes

**Fig 2:**
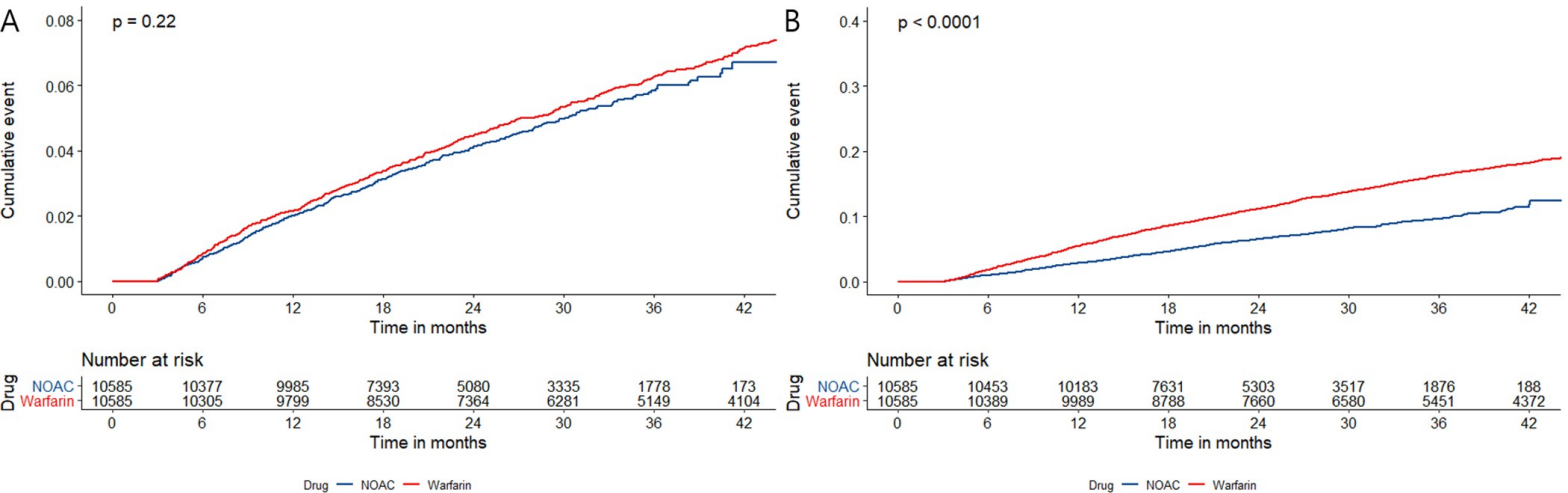
Weighted cumulative incidence curves of the primary outcomes between the NOAC and warfarin groups. A. Ischemic stroke B. All-cause mortality. NOAC, non-vitamin K antagonist oral anticoagulant.

The risk of ischemic stroke did not differ significantly between the two groups (HR, 1.05 [95% CI, 0.88–1.23], p = 0.19). The risk of all-cause mortality was also lower in the NOAC group than in the warfarin group (HR, 0.6 [95% CI, 0.52–0.69], p<0.01) (Fig 2) (S1 Table).

### Secondary outcomes

**Fig 3:**
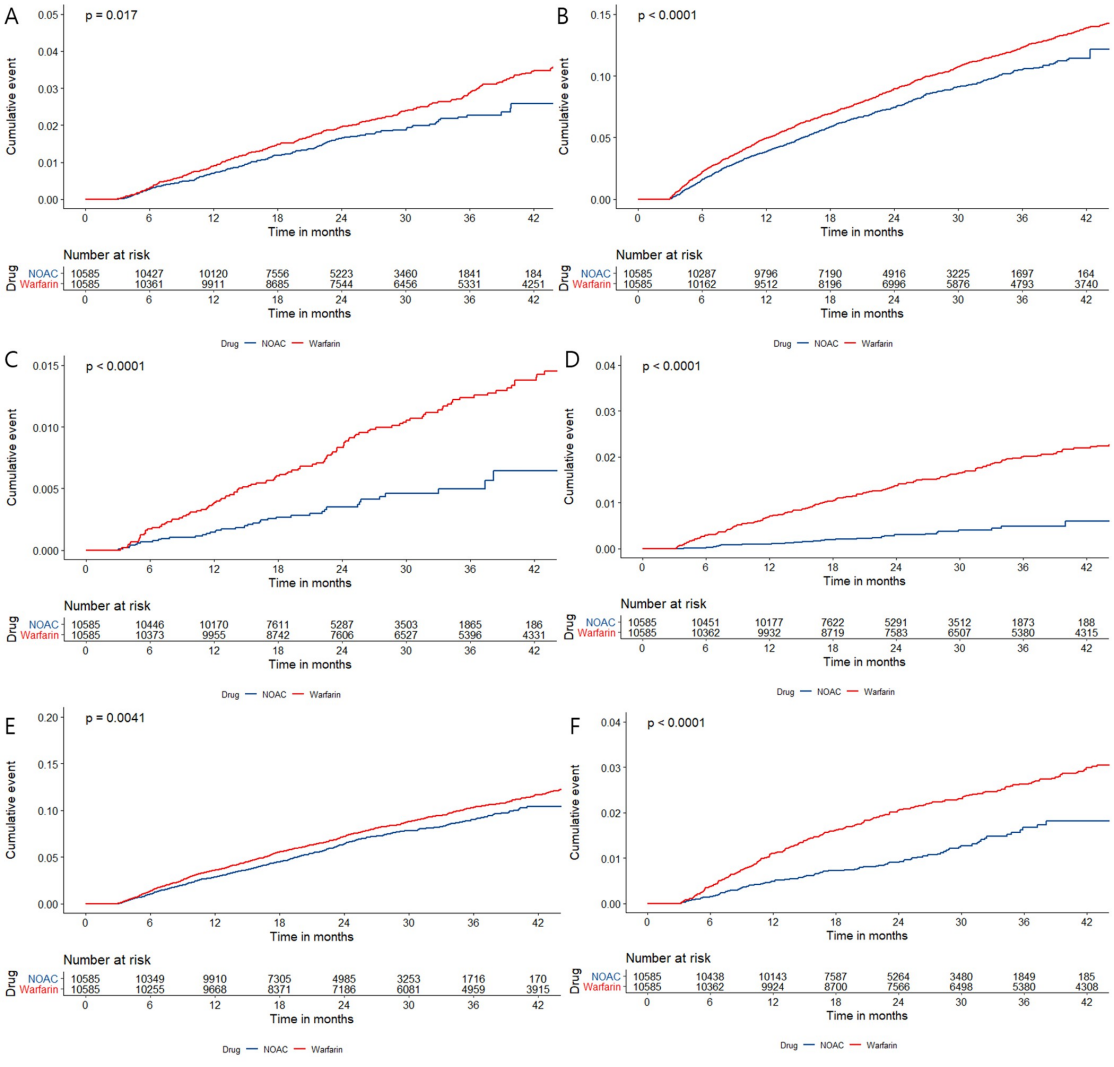
Weighted cumulative incidence curves of secondary outcomes between NOAC and warfarin groups. A. MI, B. Angina, C.CKD 4, D. ESRD, E. GI bleeding, F. ICH. NOAC, non-vitamin K antagonist oral anticoagulant; MI, Myocardial infarction; ICH, intracranial hemorrhage; GI, gastrointestinal; CV, cardiovascular.

The NOAC group had a lower risk of ICH and GI bleeding than the warfarin group (HR, 0.6 [95% CI, 0.44–0.83], p = 0.001 and HR, 0.87 [95% CI, 0.75–0.99], p = 0.04, respectively). The incidence of angina pectoris was significantly lower in the NOAC group than in the warfarin group, even after adjusting for age, sex, history of DM, history of HTN, history of heart failure, body mass index, stroke, CKD, and ESRD (HR, 0.79 [95% CI, 0.69–0.89], p<0.001). The incidence of MI was not significantly different between the groups (HR, 0.88 [95% CI, 0.66–1.17], p = 0.37). The risk of CKD and escalation to ESRD was significantly lower in the NOAC group than in the warfarin group (HR, 0.5 [95% CI, 0.28–0.88], p = 0.02 and HR, 0.16 [95% CI, 0.08–0.32], p<0.001, respectively) (S2 Table). Furthermore, NOACs were less associated with MACE (HR, 0.829 [95% CI, 0.726–0.947], p<0.01). The KM curve showed a higher event-free probability for MI, angina pectoris, CKD 4, escalation to ESRD, GI bleeding and ICH in the NOAC group than in the warfarin group (Fig 3).

**Fig 4:**
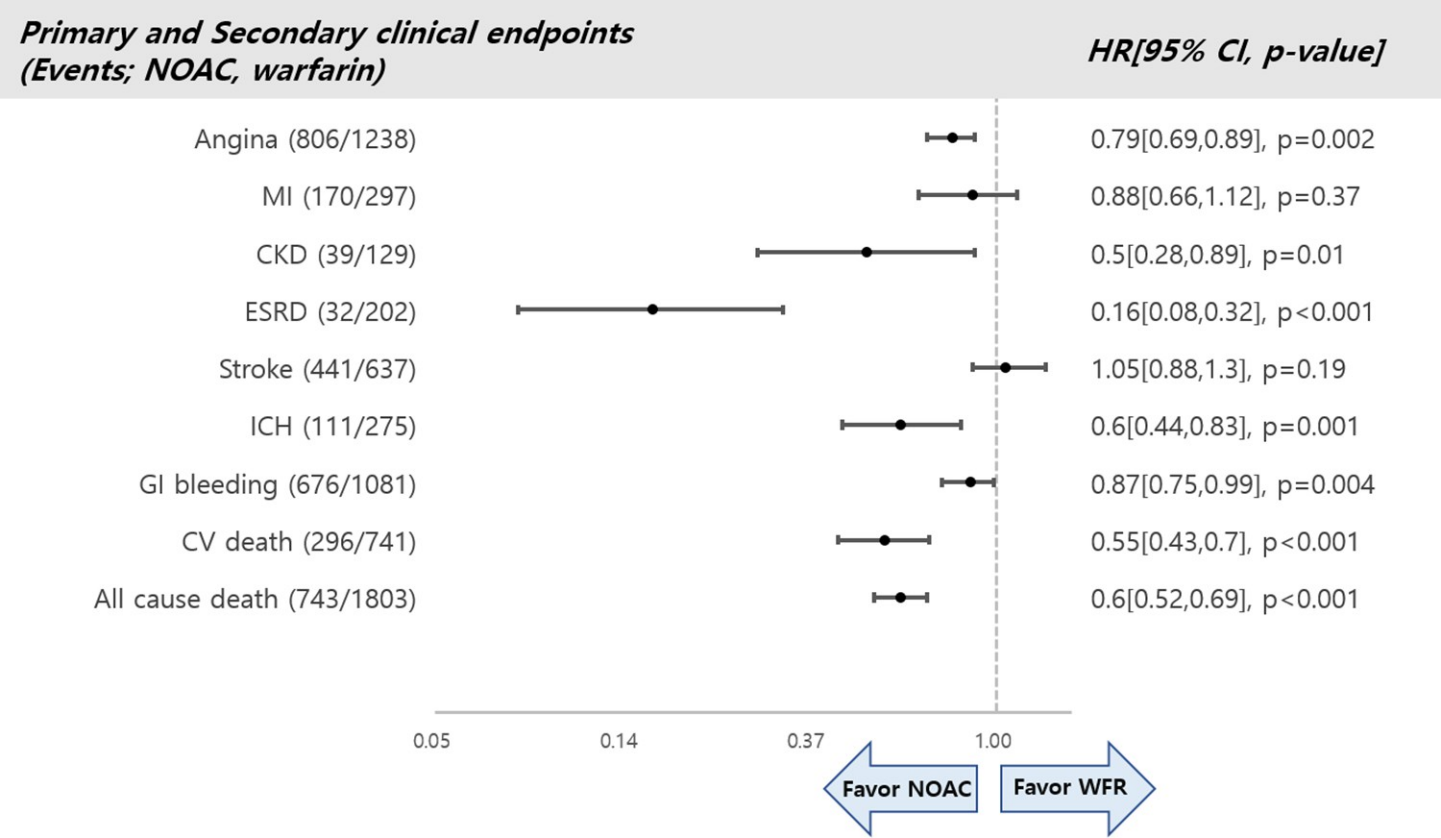
Primary and secondary outcome comparisons of NOACs and warfarin. NOAC, non-vitamin K antagonist oral anticoagulant; WFR, warfarin; MI, myocardial infarction; CKD, chronic kidney disease; ESRD, end-stage renal disease; ICH, intracranial hemorrhage; GI, gastrointestinal; CV, cardiovascular; HR, hazard ratio; CI, confidence interval.

Details relating to the primary and secondary outcomes of the warfarin and NOAC groups are shown in Fig 4.

## Discussion

Our analysis involved a large nationwide population of patients with AF who were prescribed anticoagulants in Korea. The incidence of ischemic stroke did not differ between the two groups however, incidence of all-cause mortality, angina pectoris, stage 4 CKD, and escalation to ESRD was significantly lower in the NOAC group than in the warfarin group. The outcomes for acute events, such as MI, did not significantly differ between the groups.

Furthermore, NOACs resulted in fewer bleeding events than warfarin in categories such as ICH, GI bleeding.

The effective and safe use of anticoagulation in patients with AF has posed significant challenges to physicians in clinical practice since its introduction. Based on the evidence from the Framingham Heart Study cohorts [9] and the risk stratification using the CHA_2_DS_2_-VASc score system [10], stroke prevention has become the mainstay treatment strategy in patients with AF [11]. Diverse approaches have shed light on appropriate anticoagulant selections [12]. With an increasing number of published studies favoring the use of NOACs over warfarin, NOACs appear to be the current anticoagulant of choice for patients with AF [13]. Despite substantial efforts to establish a consensus regarding NOACs and warfarin, the evidence has been conflicting. The previous studies mainly focused on the efficacy of NOACs and warfarin in preventing ischemic stroke and their safety with respect to ICH and major bleeding. Due to the high prevalence of CV comorbidities in patients with AF, it is important to assess the clinical implications of NOACs and warfarin. Warfarin, a vitamin K antagonist, has been reported to ensue vasculitis along with other vascular complications [14–19]. The use of warfarin is associated with aortic stiffness, and many researchers have investigated this in great detail and concluded that warfarin causes vitamin K deficiency, which could lead to aortic stiffness [20].

In our study, patients who were prescribed NOACs were at a lower risk of experiencing angina pectoris, stage 4 CKD, and escalation to ESRD, with a higher safety profile than those who were prescribed warfarin. Previous studies have elaborated not only on the bleeding consequences but also on the vascular complications that warfarin can cause. To the best of our knowledge, our study is the first to investigate the vascular aspects and compare specific clinical outcomes pertaining to systemic vasculature in patients using warfarin and NOACs via a nationwide large population database. Interestingly, the incidence of MI, despite showing a higher probability in the warfarin group on the KM survival curve, was not significantly different between the two groups when other risk factors were adjusted for. This may be attributed to the fundamental pathophysiological traits of the disease. MI is usually a result of an abrupt rupture of a previously formed unstable atherosclerotic plaque, causing platelet aggregation along with inflammatory response activation, resulting in total occlusion of the epicardial artery [21]. Therefore, the impact of oral anticoagulant use in MI needs further research. Although oral anticoagulants do not exert antiplatelet functions *per se*, the event itself is thrombogenic in nature and, therefore, could have influenced the outcome. Furthermore, in the current study, even after propensity score matching to minimize the baseline discrepancy between the two groups, the prevalence of stage 4 CKD, ESRD, and ICMP, were higher in the warfarin group than in the NOAC group, which could have attributed to higher incidences in the warfarin group.

Several studies have compared the efficacy and safety of warfarin and NOACs. Their primary purpose was to evaluate the ischemic and overall bleeding risks so that physicians who treat patients with AF can select the optimal anticoagulant. However, even with these validated studies, the issues of adverse vascular events and renal function deterioration remain unanswered. Recently, several studies have focused on the beneficial effects of NOACs in patients with coronary and peripheral artery diseases, as well as those with acute and chronic kidney injury.

The COMPASS trial demonstrated that rivaroxaban showed higher efficacy in patients compared with aspirin alone [22]. A similar finding was reported in patients with stable coronary disease treated with rivaroxaban [23]. Moreover, NOACs, especially dabigatran and rivaroxaban, have shown a decreased risk of poor renal outcomes compared to warfarin [24]. Our study not only reappraised the previous findings regarding safety and efficacy but also demonstrated that NOACs were associated with fewer vascular and renal events compared with warfarin. Although our study does warrant a definitive cause-and-effect relationship between warfarin and NOACs, it reminds clinicians that ischemic and bleeding risks are not the only aspects to consider when prescribing oral anticoagulants in patients with AF.

Our study had several limitations. First, this was a retrospective study that utilized a national insurance cohort. Therefore, biochemical and inflammatory markers relevant to arterial stiffness, such as the serum levels of kynurenic acid, and physiologic parameters, such as pulse wave velocity, were not included in our analysis [25]. The data were extracted from the national cohort registry. Therefore, it was not possible to obtain prescription details or socioeconomic information. If these data were accessible, a more definitive conclusion on the vascular-protective effects of NOACs and warfarin could have been achieved. Second, although patients who were prescribed NOACs showed less vascular and renal adverse events compared to patients with warfarin, the retrospective and nationwide insurance cohort nature of our study design limited our study from establishing a definitive ‘cause-and-effect’ conclusion between these two anticoagulants. Third, the study population comprised only Koreans. Therefore, larger studies with the inclusion of diverse races and ethnicities are warranted.

In conclusion, among patients with AF who needed anticoagulation therapy, patients who were prescribed NOACs showed less frequent incidences of angina pectoris, stage 4 CKD, and escalation to ESRD compared with those who were prescribed warfarin. Thus, NOACs demonstrated a higher safety profile than warfarin. Further studies to validate the causality between oral anticoagulants (NOACs and warfarin) and vascular and renal dysfunctions are needed. Such findings will enhance the understanding of oral anticoagulants among practitioners who treat patients with AF and assist with the selection of the optimal drug to benefit their patients.

## Supporting information

S1 Table Multivariate and univariate Cox regression analyses of the primary outcomes. A. Angina and MI, B. CKD stage 4 and ESRD.
(DOCX)

S2 Table Multivariate and univariate Cox regression analyses of the secondary outcomes. A. Stroke and GI bleeding, B. ICH and CV death, C. All-cause death, D. MACE.
(DOCX)
