# Supplementary material for: Cardiovascular and renal protective effects of non-vitamin K antagonist oral anticoagulants and warfarin in patients with atrial fibrillation
Source: PLoS One. 2022 Oct 13;17(10):e0275103. doi: 10.1371/journal.pone.0275103 (PMC9560050; doi:10.1371/journal.pone.0275103)
Supplement: S1 Table — A. Angina and MI, B. CKD stage 4 and ESRD. (DOCX) [file pone.0275103.s001.docx]

**Supplementary Table 1. Multivariate and univariate Cox regression analyses of the primary outcomes**

**A. Ischemic stroke and all-cause mortality**

| Primary outcomes | Ischemic stroke | | | | | | All-cause death | | | | | |
| --- | --- | --- | --- | --- | --- | --- | --- | --- | --- | --- | --- | --- |
|  | Univariate | | | Multivariate | | | Univariate | | | Multivariate | | |
| Risk factors | HR | 95% CI | p-value | HR | 95% CI | p-value | HR | 95% CI | p-value | HR | 95% CI | p-value |
| *Age* | 2.09 | 1.82-2.38 | <0.01 |  |  |  | 4.40 | 3.96-4.90 | <0.01 | 3.58 | 3.01-4.26 | <0.01 |
| *Sex* | 1.21 | 1.06-1.36 | <0.01 | 1.08 | 0.91-1.29 | <0.01 | 1.35 | 1.24-1.46 | <0.01 | 0.81 | 0.70-0.93 | <0.01 |
| *DM* | 1.25 | 1.12-1.41 | <0.01 |  |  |  | 1.55 | 1.43-1.67 | <0.01 | 1.37 | 1.19-1.57 | <0.01 |
| *HTN* | 1.51 | 1.3-1.75 | <0.01 | 1.42 | 1.13-1.78 | 0.04 | 1.73 | 1.57-1.92 | <0.01 |  |  |  |
| *HF* | 1.31 | 1.13-1.47 | <0.01 | 1.04 | 0.86-1.24 | <0.01 | 1.55 | 1.43-1.68 | <0.01 | 1.45 | 1.26-1.66 | <0.01 |
| *Stroke* | 0.08 | 0.05-0.12 | <0.01 |  |  |  | 1.56 | 1.43-1.70 | <0.01 | 1.54 | 1.32-1.78 | <0.01 |
| *CKD* | 1.55 | 0.88-2.73 | 0.13 | 0.67 | 0.25-1.82 | <0.01 | 2.24 | 1.65-3.05 | <0.01 | 1.62 | 1.03-2.53 | 0.04 |
| *ESRD* | 1.69 | 1.27-2.27 | <0.01 |  |  |  | 2.87 | 2.47-3.34 | <0.01 | 2.93 | 2.22-3.86 | <0.01 |
| *BMI* | 1.12 | 0.94-1.33 | 0.19 | 1.12 | 0.94-1.33 | 0.09 | 1.93 | 1.68-2.22 | <0.01 | 1.74 | 1.51-2.00 | <0.01 |
| *NOAC* | 0.92 | 0.82-1.05 | 0.21 |  |  |  | 0.58 | 0.53-0.63 | <0.01 | 0.60 | 0.52-0.70 | <0.01 |

HR, hazard ratio; CI, confidence interval; DM, diabetes mellitus; HTN, hypertension; HF, heart failure; CKD, chronic kidney disease; ESRD, end-stage renal disease; BMI, body mass index; NOAC, non-vitamin K antagonist oral anticoagulant
