# Supplementary material for: Cardiovascular and renal protective effects of non-vitamin K antagonist oral anticoagulants and warfarin in patients with atrial fibrillation
Source: PLoS One. 2022 Oct 13;17(10):e0275103. doi: 10.1371/journal.pone.0275103 (PMC9560050; doi:10.1371/journal.pone.0275103)
Supplement: S2 Table — A. Stroke and GI bleeding, B. ICH and CV death, C. All-cause death, D. MACE. (DOCX) [file pone.0275103.s002.docx]

**Supplementary Table 2. Multivariate and univariate Cox regression analyses of the secondary outcomes**

**A. MI and Angina**

| Secondary outcomes | MI | | | | | | Angina | | | | | |
| --- | --- | --- | --- | --- | --- | --- | --- | --- | --- | --- | --- | --- |
|  | Univariate | | | Multivariate | | | Univariate | | | Multivariate | | |
| Riskf factors | HR | 95% CI | p-value | HR | 95% CI | p-value | HR | 95% CI | p-value | HR | 95% CI | p-value |
| *Age* | 2.15 | 1.76-2.63 | <0.01 | 1.8 | 1.33-2.44 | <0.01 | 1.06 | 0.97-1.16 | 0.16 |  |  |  |
| *Sex* | 1.25 | 1.04-1.50 | <0.01 |  |  |  | 1.1 | 1.01-1.2 | 0.03 |  |  |  |
| *DM* | 1.5 | 1.25-1.80 | <0.01 |  |  |  | 0.99 | 0.91-1.08 | 0.82 |  |  |  |
| *HTN* | 1.46 | 1.17-1.83 | <0.01 |  |  |  | 0.97 | 0.88-1.07 | 0.53 |  |  |  |
| *HF* | 1.31 | 1.09-1.58 | <0.01 |  |  |  | 0.89 | 0.82-0.98 | 0.02 |  |  |  |
| *Stroke* | 1.28 | 1.03-1.59 | 0.02 |  |  |  | 0.94 | 0.84-1.06 | 0.3 |  |  |  |
| *CKD* | 2.4 | 1.19-4.83 | 0.01 | 2.57 | 1.12-5.89 | 0.02 | 1.02 | 0.61-1.69 | 0.94 |  |  |  |
| *ESRD* | 2.75 | 1.92-3.98 | <0.01 |  |  |  | 1.62 | 1.29-2.01 | <0.01 | 1.5 | 1.01-2.19 | 0.04 |
| *BMI* | 0.95 | 0.73-1.25 | 0.75 |  |  |  | 1.02 | 0.91-1.16 | 0.65 |  |  |  |
| *NOAC* | 0.78 | 0.65-0.95 | 0.02 |  |  |  | 0.83 | 0.76-0.91 | <0.01 | 0.8 | 0.69-0.89 | <0.01 |

MI, myocardial infarction; HR, hazard ratio; CI, confidence interval; DM, diabetes mellitus; HTN, hypertension; HF, heart failure; CKD, chronic kidney disease; ESRD, end-stage renal disease; BMI, body mass index; NOAC, non-vitamin K antagonist oral anticoagulant

**B. CKD 4 and ESRD**

| Secondary outcomes | CKD 4 | | | | | | ESRD | | | | | |
| --- | --- | --- | --- | --- | --- | --- | --- | --- | --- | --- | --- | --- |
|  | Univariate | | | Multivariate | | | Univariate | | | Multivariate | | |
| Riskf factors | HR | 95% CI | p-value | HR | 95% CI | p-value | HR | 95% CI | p-value | HR | 95% CI | p-value |
| *Age* | 3.14 | 2.17-4.56 | <0.01 | 2.43 | 1.32-4.44 | <0.01 | 2.96 | 2.17-4.01 | <0.01 | 3 | 1.70-5.20 | <0.01 |
| *Sex* | 1.43 | 1.05-1.93 | 0.022 |  |  |  | 1.28 | 0.98-1.65 | <0.01 |  |  |  |
| *DM* | 2.67 | 1.95-3.66 | <0.01 | 2.2 | 1.31-3.71 | <0.01 | 2.8 | 2.14-3.65 | <0.01 | 1.7 | 1.13-2.67 | 0.01 |
| *HTN* | 4.13 | 2.39-7.14 | <0.01 |  |  |  | 3.44 | 2.23-5.29 | <0.01 |  |  |  |
| *HF* | 2.09 | 1.54-2.83 | <0.01 | 1.89 | 1.16-3.04 | <0.01 | 1.83 | 1.41-2.36 | <0.01 |  |  |  |
| *Stroke* | 1.38 | 0.974-1.96 | 0.07 |  |  |  | 1.65 | 1.24-2.19 | <0.01 |  |  |  |
| *CKD* | 9.06 | 4.78-17.16 | <0.01 | 10.1 | 4.98-20.63 | <0.001 | 42.65 | 30.85-58.95 | <0.01 | 18 | 10.67-29.14 | <0.01 |
| *ESRD* | 3.43 | 1.99-5.94 | <0.01 | 4.22 | 1.86-9.62 | <0.01 | 0.32 | 0.07-1.28 | 0.11 |  |  |  |
| *BMI* | 0.99 | 0.62-1.58 | 0.96 |  |  |  | 1.12 | 0.75-1.68 | 0.56 |  |  |  |
| *NOAC* | 0.42 | 0.29-0.62 | <0.01 | 0.5 | 0.28-0.88 | <0.01 | 0.22 | 0.15-0.32 | <0.01 | 0.2 | 0.07-0.32 | <0.01 |

HR, hazard ratio; CI, confidence interval; DM, diabetes mellitus; HTN, hypertension; HF, heart failure; CKD, chronic kidney disease; ESRD, end-stage renal disease; BMI, body mass index; NOAC, non-vitamin K antagonist oral anticoagulant

**C. ICH and GI bleeding**

| Secondary outcomes | ICH | | | | | | GI | | | | | |
| --- | --- | --- | --- | --- | --- | --- | --- | --- | --- | --- | --- | --- |
|  | Univariate | | | Multivariate | | | Univariate | | | Multivariate | | |
| Riskf factors | HR | 95% CI | p-value | HR | 95% CI | p-value | HR | 95% CI | p-value | HR | 95% CI | p-value |
| *Age* | 2.23 | 1.78-2.79 | <0.01 | 2.21 | 1.58-3.11 | <0.01 | 1.48 | 1.34-1.63 | <0.01 | 1.27 | 1.11-1.46 | <0.01 |
| *Sex* | 1.32 | 1.09-1.62 | <0.01 |  |  |  | 1.02 | 0.92-1.12 | 0.67 | 0.86 | 0.74-0.99 | 0.04 |
| *DM* | 1.09 | 0.89-1.33 | 0.38 |  |  |  | 1.21 | 1.11-1.33 | <0.01 |  |  |  |
| *HTN* | 1.59 | 1.23-2.06 | <0.01 |  |  |  | 1.35 | 1.2-1.52 | <0.01 | 1.21 | 1.03-1.43 | 0.02 |
| *HF* | 1.02 | 0.82-1.26 | 0.85 |  |  |  | 1.11 | 1.01-1.22 | 0.03 |  |  |  |
| *Stroke* | 1.67 | 1.34-2.09 | <0.01 | 1.68 | 1.2-2.34 | <0.01 | 1.09 | 0.98-1.23 | 0.11 |  |  |  |
| *CKD* | 2.15 | 0.96-4.83 | 0.06 |  |  |  | 1.77 | 1.16-2.68 | <0.01 | 1.71 | 1.00-2.91 | 0.05 |
| *ESRD* | 3.01 | 2.06-4.39 | <0.01 | 2.75 | 1.42-5.3 | <0.01 | 2.46 | 2.02-3.01 | <0.01 | 2.03 | 1.41-2.94 | <0.01 |
| *BMI* | 1.47 | 1.09-1.98 | 0.01 |  |  |  | 1.03 | 0.91-1.18 | 0.59 |  |  |  |
| *NOAC* | 0.53 | 0.42-0.67 | <0.01 | 0.6 | 0.44-0.83 | <0.01 | 0.86 | 0.78-0.95 | <0.01 | 0.86 | 0.75-0.99 | 0.04 |

ICH, intracranial hemorrhage; GI. Gastrointestinal; HR, hazard ratio; CI, confidence interval; DM, diabetes mellitus; HTN, hypertension; HF, heart failure; CKD, chronic kidney disease; ESRD, end-stage renal disease; BMI, body mass index; NOAC, non-vitamin K antagonist oral anticoagulant

**D. MACE**

| Secondary outcomes | MACE | | | | | |
| --- | --- | --- | --- | --- | --- | --- |
|  | Univariate | | | Multivariate | | |
| Risk factors | HR | 95% CI | p value | HR | 95% CI | p value |
| *Age* | 1.05 | 1.05-1.05 | <0.01 | 1.04 | 1.04-1.05 | <0.01 |
| *Sex* | 1.43 | 1.32-1.55 | <0.01 |  |  |  |
| *DM* | 1.31 | 1.21-1.42 | <0.01 | 1.14 | 1.00-1.29 | 0.04 |
| *HTN* | 1.74 | 1.54-1.93 | <0.01 | 1.25 | 1.05-1.48 | 0.01 |
| *HF* | 1.52 | 1.40-1.65 | <0.01 | 1.32 | 1.15-1.50 | <0.01 |
| *Stroke* | 0.89 | 0.80-0.99 | <0.01 | 0.68 | 0.57-0.82 | <0.01 |
| *CKD* | 1.89 | 1.34-2.68 | <0.01 |  |  |  |
| *ESRD* | 1.95 | 1.62-2.34 | <0.01 | 1.79 | 1.26-2.54 | <0.01 |
| *BMI* | 1.26 | 1.12-1.43 | <0.01 | 1.17 | 1.03-1.33 | 0.01 |
| *NOAC use* | 0.74 | 0.68-0.81 | <0.01 | 0.83 | 0.72-0.94 | <0.01 |

MACE, Major adverse cardiovascular event; HR, hazard ratio; CI, confidence interval; DM, diabetes mellitus; HTN, hypertension; HF, heart failure; CKD, chronic kidney disease; ESRD, end-stage renal disease; BMI, body mass index; NOAC, non-vitamin K antagonist oral anticoagulant
